# IDH mutation, 1p19q codeletion and ATRX loss in WHO grade II gliomas

**DOI:** 10.18632/oncotarget.4497

**Published:** 2015-07-03

**Authors:** Heather E. Leeper, Alissa A. Caron, Paul A. Decker, Robert B. Jenkins, Daniel H. Lachance, Caterina Giannini

**Affiliations:** ^1^ Neuro-Oncology, Advocate Medical Group, Park Ridge, IL 60068, USA; ^2^ Experimental Pathology, Mayo Clinic SW, Rochester, MN 55905, USA; ^3^ Biomedical Statistics and Informatics, Mayo Clinic SW, Rochester, MN 55905, USA; ^4^ Neurology, Mayo Clinic SW, Rochester, MN 55905, USA; ^5^ Anatomic Pathology, Mayo Clinic SW, Rochester, MN 55905, USA

**Keywords:** diffuse gliomas, WHO grade II, IDH mutation, ATRX, 1p19q codeletion

## Abstract

**Background:**

Epigenetic, genetic, and molecular studies have identified several diagnostic and prognostic markers in diffuse gliomas. Their importance for evaluating WHO grade II gliomas has yet to be specifically delineated.

**Methods:**

We analyzed markers, including IDH mutation(IDHmut), 1p19q codeletion(1p19qcodel), ATRX expression loss(ATRX loss) and p53 overexpression, and outcomes in 159 patients with WHO grade II oligodendroglioma, oligoastrocytoma, and astrocytoma (2003–2012).

**Results:**

IDHmut was found in 141(91%) and ATRX loss in 64(87%) of IDHmut-noncodel tumors (*p* = 0.003). All codeleted tumors (*n* = 66) were IDHmut. Four subgroups were identified: IDHmut-codel, 66(43%); IDHmut-noncodel-ATRX loss, 60(39%); IDHmut-noncodel-ATRXwt, 9(6%); IDHwt, 14(9%). Median survival among 4 groups was significantly different (*p* = 0.038), particularly in IDHmut-codel (median survival 15.6 years) compared to the remaining 3 groups (*p* = 0.025). Survival by histology was not significant. Overall (OS), but not progression-free (PFS), survival was significantly longer with gross total resection vs. biopsy only (*p* = 0.042). Outcomes for patients with subtotal resection were not significantly different from those with biopsy only. Among these uniformly treated patients, OS far exceeds PFS, particularly in those with 1p/19q codeletion.

**Conclusions:**

For WHO grade II diffuse glioma, molecular classification using 1p/19qcodel, IDHmut, and ATRX loss more accurately predicts outcome and should be incorporated in the neuropathologic evaluation.

## INTRODUCTION

The diagnosis of “lower-grade” diffusely infiltrative glioma (WHO grade II and III) is a histologic diagnosis largely based upon neoplastic cell morphology and the presence or absence of features of anaplasia (e.g. mitoses, microvascular proliferation, and necrosis), according to the 2007 WHO Classification of Tumours of the Central Nervous System [1]. Tumors with oligodendroglial and/or astrocytic morphologies are classified as oligodendroglioma (O), oligoastrocytoma (OA), and astrocytoma (A), respectively, and graded as either II or III. There is considerable interobserver variability in assessing both morphology and grade, which has significant clinical implications [2]. In particular, the diagnosis of grade II is extremely important for current management approaches. Unfortunately, molecular diagnostic information for this strictly defined group has not been clearly delineated in recent studies, which have combined the entire spectrum of grade II and grade III gliomas [3]. In recent years, there have been major advances in our understanding of the epigenetic, genetic, and molecular alterations in glioma [4–6]. Isocitrate dehydrogenase (IDH) gene mutations, estimated to occur in 70% to 90% of diffuse lower-grade gliomas, affect the epigenetic regulation of the genome and are strongly implicated in both tumorigenesis and prognosis [3, 7–11]. Secondary analysis of clinical trial RTOG 9402 retrospectively validated IDH mutation as both a prognostic and a predictive marker of response to PCV (procarbazine, lomustine, and vincristine) chemotherapy and radiation independent of 1p19q codeletion (1p19q codel) [12, 13]. Loss of heterozygosity of 1p19q, occurring in 60% to 80% of O and up to 45% of OA [14], is an established genetic marker of glioma with predominant oligodendroglial morphology [15]. Codeletion of 1p19q has also been demonstrated to be a prognostic marker [13] as well as predictive of responsiveness to PCV chemotherapy and radiation [16, 17]. Inactivating mutations of alpha-thalassemia/mental retardation syndrome X-linked (ATRX) gene, correlated with the alternative lengthening of telomeres (ALT) phenotype, are highly associated with IDH and TP53 mutations but mutually exclusive with 1p19q codel [12, 18]. Prevalence of ATRX loss, which in some studies has been associated with favorable prognosis in anaplastic gliomas, ranges from 33% to 72% of IDH mutated noncodel gliomas [18, 19]. Mutation of the tumor suppressor gene TP53, frequently resulting in p53 protein overexpression, is also mutually exclusive with 1p19q codel [20, 21]. Approximately 60% of diffuse A and 40% of OA carry TP53 mutation [20].

Here, we report the frequency of IDH mutations, 1p19q codeletion, ATRX expression loss, and p53 overexpression in strictly defined WHO grade II O, OA, and A, treated at the Mayo Clinic over a 10-year period (2003–2012) and define their diagnostic and prognostic value.

## RESULTS

### Clinical data

Patient characteristics, treatment history, and pathologic data are summarized in Table 1. The median age of all patients was 37 years (interquartile range 29–46), ranging from 16 to 84 years of age. There were 70 females and 89 males. The median follow-up in subjects not known to be deceased is 4 years (range: 4 days to 23.6 years). A total of 31 patients were known to be deceased due to tumor. The majority (87 of 159) (55%) had >90% resection of tumor as defined by T2/FLAIR hyperintensity, while 50 (31%) had subtotal resection, and 22 (14%) had stereotactic biopsy only. Most patients (81 of 159) (57%) were observed after initial surgery, and 54 (34%) had radiation without chemotherapy after initial surgery. Only 14 (9%) patients received upfront chemotherapy, 12 of whom also received radiation.

**Table 1:** Patient clinical data, treatment history and pathology

Patient Characteristics	All Patients *n* = 159 (%)	IDHmut *n* = 141 (%)	Codel *n* = 69 (%)	ATRX loss *n* = 64 (%)	IDHwt *n* = 14 (%)
**Age, in years**: ≤ 40	98 (62)	90 (64)	40 (58)	44 (69)	4 (29)
>40	61 (38)	51 (36)	29 (42)	20 (31)	10 (71)
**Sex**: Female	69 (43)	59 (42)	40 (58)	18 (28)	6 (43)
Male	90 (57)	82 (58)	29 (42)	46 (72)	7 (50)
**Location**: Frontal only^a^	89 (56)	85 (60)	46 (67)	35 (55)	2 (14)
Multiple lobes^b^	21 (13)	17 (12)	8 (12)	9 (14)	4 (29)
Insula^c^	11 (7)	8 (6)	4 (6)	6 (9)	0 (0)
Temporal only	17 (11)	11 (7)	1 (0)	8 (12)	7 (50)
Parietal/Parieto-occipital	20 (13)	18 (13)	10 (15)	7 (10)	1 (7)
Brainstem	1 (0)	1 (0)	0 (0)	0 (0)	0 (0)
**Presented with seizure**	113 (71)	98 (70)	50 (73)	43 (67)	11 (79)
**Surgery**: >90% resected	87 (55)	80 (57)	41 (59)	31 (48)	6 (43)
Subtotal	50 (31)	42 (30)	16 (23)	27 (42)	7 (50)
Biopsy	22 (14)	19 (13)	12 (17)	6 (9)	1 (7)
**Post-op treatment**: Observation	91 (57)	81 (57)	43 (62)	31 (48)	9 (64)
RT alone	54 (34)	48 (34)	20 (29)	27 (42)	4 (29)
RT + adjuvant chemo	4 (3)	3 (2)	2 (3)	2 (3)	0 (0)
RT/TMZ + adj TMZ	8 (5)	7 (5)	2 (3)	4 (6)	1 (7)
Chemo alone	2 (1)	2 (1)	2 (3)	0 (0)	0 (0)
**Diagnosis**: Astrocytoma	21 (13)	13 (9)	2 (3)	11 (17)	8 (57)
Oligodendroglioma	49 (31)	42 (30)	36 (52)	7 (11)	4 (29)
Oligoastrocytoma	89 (56)	86 (61)	31 (45)	46 (72)	2 (14)
**p53 expression**:					
0–10%	71 (45)	65 (46)	50 (72)	12 (18)	5 (36)
11–50%	34 (21)	28 (20)	12 (17)	15 (23)	6 (43)
51–80%	38 (24)	35 (25)	0 (0)	29 (45)	3 (21)
>80%	3 (2)	3 (2)	0 (0)	3 (5)	0 (0)

a Frontal lobe only, excluding insula

b Multiple lobes involved including frontal defined as frontal lobe involved plus at least one adjacent lobe excluding the insula

c Insula with possible involvement of adjacent frontal and/or temporal lobe

#### Molecular marker results

**Figure 1:**
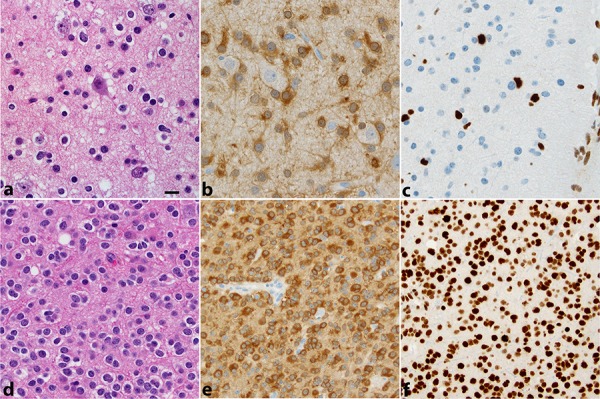
Two WHO grade II gliomas are illustrated The first **a.** diagnosed as oligoastrocytoma displays infiltration of cortex by a low-density population of cells, in part, with round regular nuclei and clear halos (“oligodendroglial”), in part, with irregular elongated nuclei (“astrocytic”). The cells are IDH1R132H positive **b.** and lack ATRX expression **c.** In the second **d.** diagnosed as oligodendroglioma, tumor cells are IDH1R132H positive **e.** and maintain ATRX expression **f.** This second tumor showed 1p19q codeletion by FISH. All photos were taken at 400 × (magnification bar shown in panel a 10 microns).

**Figure 2:**
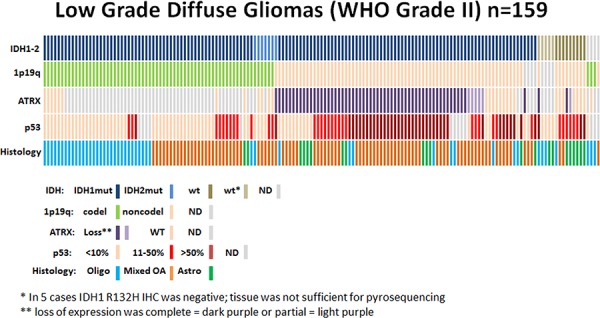
Low grade diffuse gliomas Data regarding IDH status, 1p19q codeletion, ATRX status, p53 expression, and histological diagnosis are summarized for all patients (*n* = 159). Each column represents a patient.

The results of the IDH, 1p19q, ATRX testing are summarized in Figures 1 and 2 as well as Table 1.

**IDH mutations**: An IDH mutation was present in 141 of 155 (91%): 127 (90%) R132H mutations, 14 (10%) rare mutations including 2 R132C, 2 R132G, 1 R132S, 2 R132 unknown, 3 R172K, 1 R172H, and 3 R172 unknown mutations. Of 14 negative cases, 5 were tested only by IDH1R132H IHC due to insufficient tissue, and 9 were negative by both IHC and IDH1-IDH2 pyrosequencing. In the last 4 (of 159) tumors, sufficient tissue for testing was unavailable.

**1p19q codeletion**: Codeletion of 1p19q was present in 69 of 151 (46%) tumors tested: 2 of 18 (11%) A; 31 of 86 (36%) OA; and 36 of 47 (77%) O. Of the remaining 8 (of 159) tumors, 2 failed testing and 6 had insufficient tissue. All 1p19q codel tumors (*n* = 69) were IDH mutant (other than 3 tumors in which IDH status could not be determined).

**ATRX loss**: ATRX expression was lost in 61 of 70 (87%) IDHmut-noncodel tumors and 3 of 14 (21%) IDHwt tumors. Among the histologic diagnoses, ATRX expression was lost in 11 of 16 (69%) of A, 46 of 60 (77%) of OA, and 7 of 17 (41%) of O. None of the 12 IDHmut-codel tumors tested had ATRX loss. Nine cases had either insufficient tissue (*n* = 6) or failed testing (*n* = 3).

**Expression of p53**: Among the 66 IDHmut-codel tumors tested, p53 expression was limited to <10% of the cells in 48 (79%); and in the remaining 13 (21%), p53 expression was <50%. Of the 55 IDHmut-noncodel-ATRXloss tumors tested, 31 (56%) had p53 expression >50%. Indeed, the only 3 tumors with p53 expression >80% were also IDH mut-noncodel-ATRX loss. Among the 9 IDHmut-noncodel-ATRXwt tumors, p53 expression was <10% in 2 (22%), < 50% in 2 (22%), and >50% in 5 (56%). Of the 14 IDHwt tumors, p53 expression was <10% in 5 (36%), <50% in 6 (43%), and >50% in 3 (21%).

Four subgroups were identified: IDHmut-codel in 66 of 155 (43%), IDHmut-noncodel-ATRX loss in 60 (39%), IDHmut-noncodel-ATRXwt in 9 (6%), and 14 IDHwt (9%) (Figure 2). All histological diagnoses were found in each of the four subgroups, although O was most frequent among IDHmut-codel [33 of 66 (50%)], OA among IDHmut-ATRX loss [46 of 61 (75%)], and A among IDHwt [8 of 14 (57%)]. Among the 37 previously published [4], 17 were IDHmut-codel, 16 were IDHmut-noncodel-ATRX loss, and 4 were IDHmut-noncodel-ATRXwt. The median age of patients with IDH mutation was 36 years of age compared to 44 years of age for those with IDHwt tumors (*p* = 0.0554). There were no significant age differences among the remaining subgroups.

### Clinical outcome

These patients presented with seizures 71% of the time, regardless of molecular group or tumor location. Frontal lobe presentation in isolation at the poles or convexity, or also involving insula or temporal lobe, occurred in 110 (78%) in those with IDHmut, 58 (84%) of those with 1p/19q codel, but only 6 (46%) IDHwt. Conversely, tumor restricted to the temporal lobe almost never occurs in 1p/19q codel patients but occurred in 7 (50%) of those IDHwt. Parieto-occipital lobes were involved primarily in 18 (13%) of IDHmut cases and less frequently (*n* = 1, 7%) in IDHwt cases. Despite these differences, in our fairly uniform neurosurgical practice inclined to maximally safe resection, there was no statistically significant difference in extent of resection achieved between any of the molecular groups. Specific pairwise comparisons of tumor locations and extent of resection by location between groups was not possible because of small numbers in some groups.

**Figure 3:**
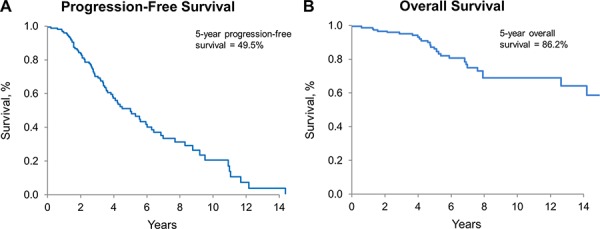
Progression-free and overall survival for all patients **A.** PFS for all patients, estimated by Kaplan-Meier method. 5-year PFS: 49.5% (95% CI: 40.6%–60.4%). **B.** OS for all patients, estimated by Kaplan-Meier method; 5-year OS: 86.2% (95% CI: 79.4%–93.5%)

**Figure 4:**
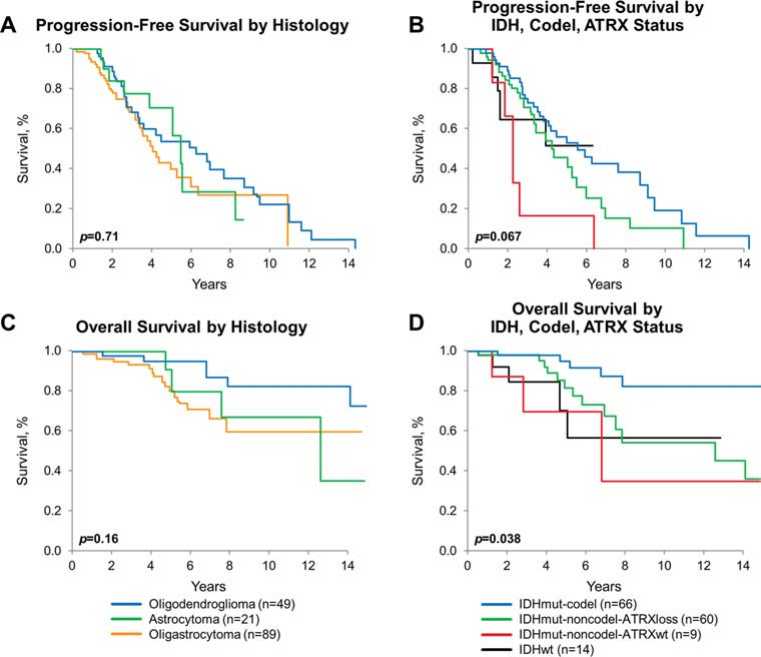
Progression-free and overall survival by diagnosis and molecular subgroups **A.** PFS by histologic diagnosis, estimated by Kaplan-Meier method, *p* = 0.71. **B.** OS by histologic diagnosis, estimated by Kaplan-Meier method, *p* = 0.16. **C.** PFS by molecular subgroup [IDHmut-codel (*n* = 65), IDHmut-noncodel-ATRXloss (*n* = 59), IDHmut-noncodel-ATRXwt (*n* = 9), IDHwt (*n* = 14)], estimated by Kaplan-Meier method, *p* = 0.067. **D.** OS by molecular subgroup [IDHmut-codel (*n* = 66), IDHmut-noncodel-ATRXloss (*n* = 60), IDHmut-noncodel-ATRXwt (*n* = 9), IDHwt (*n* = 14)], estimated by Kaplan-Meier method, *p* = 0.038. All *p*-values adjust for age, sex, histomorphologic diagnosis, extent of resection, and treatment.

The median PFS and the median overall survival (OS) for the collective cohort is 5 years and 15.3 years, respectively (Figure 3A, 3B). There was no difference in either PFS or OS among the three histologies by Kaplan-Meier method, adjusted for patient and treatment characteristics (Figure 4A and 4C). There was a significant difference in OS (*p* = 0.038) but not in PFS (*p* = 0.067), comparing the four molecular subgroups, adjusting for age, gender, EOR, histologic diagnosis, and treatment (Figure 4B, 4D). Due to insufficient sample size, we lacked statistical power to test for significant differences in pairwise comparison. The IDHmut-codel subgroup had the longest median PFS (5.6 years) and OS (15.3 years); and OS was significantly longer compared to the other three molecular groups combined (*p* = 0.025). The IDHmut-noncodel-ATRX loss subgroup median PFS was 4.4 years, and median OS was 12.7 years compared to the IDHmut-noncodel-ATRXwt subgroup median PFS of 2.2 years and median OS of 6.9 years. Median PFS and OS for the IDHwt subgroup were not achieved; however, the 5-year estimate for PFS is 51% (95% CI: 29%–93%), and OS is 71% (95% CI: 47%–100%).

**Figure 5:**
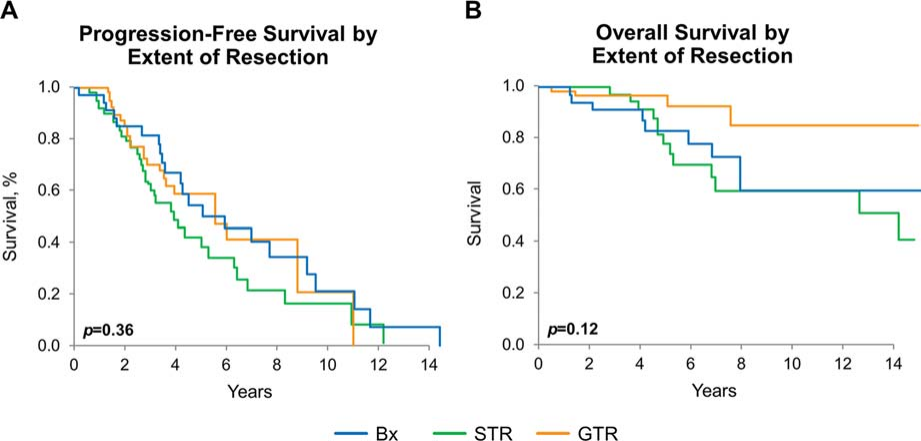
Progression-free and overall survival by extent of resection **A.** PFS by extent of resection estimated by Kaplan-Meier method, *p* = 0.36. **B.** OS by extent of resection, estimated by Kaplan-Meier method: entire cohort *p* = 0.12, pairwise GTR vs. biopsy-only: *p* = 0.042. *p*-values adjust for age, sex, histomorphologic diagnosis, and treatment.

For the entire cohort, OS, but not PFS, was significantly improved for those who had gross total resection (GTR) when compared to those who had only biopsy (*p* = 0.042). Outcome for subtotal resection was not significantly different from those with biopsy only (Figure 5A and 5B). Findings were similar when the analysis was limited to any IDH mutant tumor (data not shown). Sample size precluded testing outcome for each molecular group stratified by EOR.

## DISCUSSION

The WHO classification of diffuse gliomas is based on a wealth of clinicopathologic data; however, subjectivity complicates its uniform application. With long-term outcome data from EORTC 26951 and RTOG 9402, the predictive and prognostic significance of 1p19q codel as well as IDH mutation is established in anaplastic gliomas [16, 17]. Recently, ATRX, involved in the regulation of telomeres, was also found to be prognostically important in anaplastic gliomas [19]. Overexpression of p53 is commonly used in clinical practice, although its correlation with TP53 mutation status is far from perfect [21]. The value of these three markers in strictly defined grade II gliomas has yet to be fully established [22, 23]. Towards this end, by using techniques commonly available in clinical laboratories, we analyzed the frequency of alterations in IDH, 1p19q, ATRX, and p53 expression in a uniformly managed cohort of Mayo Clinic patients with strictly defined WHO grade II diffuse gliomas and compared clinical outcomes adjusted for patient and treatment factors.

We chose to test for IDH mutation first by IHC, followed by pyrosequencing in IHC-negative cases as this test sequence is very efficient in clinical practice. IDH1R132H IHC not only allows rapid detection of the most common mutation of IDH, accounting for approximately 90% of the IDH mutations, but in addition can detect even single tumor cells below the density threshold necessary for IDH1-IDH2 pyrosequencing [24]. In this series, all tumors were tested for 1p19q codeletion by FISH, which has been, up to now, widely popular in most clinical laboratories. Evidence has accumulated that FISH is an imperfect technique to test for 1p19q codeletion with a risk of false-positive results as it may not distinguish between partial/interstitial deletions and whole-arm deletions as the length of DNA covered by FISH probes is limited [25], a phenomenon which, in our experience, is uncommon and most frequently seen in high-grade gliomas. In the future, it is likely that laboratories will use techniques for 1p/19q analysis that more extensively ‘scan’ for presence/absence of loss of the relevant chromosome arms. In our own clinical practice, we are in the process of converting the assessment of 1p19q from FISH to aCGH.

Previous investigations of the frequencies of molecular and genetic alterations in low-grade gliomas disclosed IDH mutations in as many as 85% [14], 1p19q codeletion in up to 80% of O [26], and ATRX loss as high as 70% to 72% in IDH mut-noncodel tumors [12, 18]. We identified in 21 A, 49 O, and 89 OA alteration rates of 91% in IDH, 46% in 1p19q loss, and among IDHmut-noncodel tumors, 85% prevalence of ATRX loss. No instance of ATRX loss was found in the 12 of 66 IDHmut-codel tumors tested, and only 7 of the 64 ATRX loss tumors were O, consistent with prior correlations of ATRX loss with astrocytic and oligoastrocytic morphology [20, 21]. The frequency of ATRX loss in 3 (of 14) IDHwt tumors (21%), 2% of the entire cohort, is similar to previously reported ATRX loss in tumors which are not IDHmut-noncodel [19].

Our study confirms that ATRX loss is highly correlated with IDH mutation. The high rate of ATRX alteration in our strictly defined grade II IDHmut-noncodel tumor cohort supports the use of ATRX as a discriminating molecular marker in clinical practice, together with IDH testing.

We demonstrate that IDH mutation, 1p19q codeletion, and ATRX loss, considered together, may better define the clinical course than histologic diagnosis alone, after adjusting for age, EOR, and treatment. While better prognosis of patients with IDH mutations and 1p19q codeletion is well-established [3, 12, 16, 17], ATRX loss has only recently been of clinical research interest. Several studies have reported ATRX loss in grade II and III IDHmut-noncodel tumors to be prognostically favorable [18, 19]. Conversely, in this series of strictly defined grade II gliomas, IDHwt and IDHmut-non-codel-ATRXwt subgroups do somewhat more poorly, particularly in terms of relatively short PFS, but OS is much better than glioblastoma, as might be surmised from combined grade II and grade III glioma cohorts [27]. It is likely that additional studies will define markers more capable of identifying the most aggressive grade II lesions yet, even within the four groups we have identified.

There was no difference in frequency of seizure presentation between the groups. There was no difference in EOR achieved between any of the molecular groups. There are differences in the location frequencies between the groups [27], but location did not influence the extent of resection. In this cohort of relatively young patients with minimal comorbidities, the majority of whom were operated in a surgical practice inclined toward maximally safe resections incorporating image-guided techniques and awake craniotomy, extent of resection can be confidently assumed to be related to tumor volume and/or involvement in eloquent brain. Realizing these confounders, we confirm observations from other series that GTR (in this series, meaning >90% of all MRI defined T2 signal abnormality) resulted in improved survival, at least when compared to biopsy alone [28, 29]. However, having shown that extent of resection was not significantly different between the molecular groups, yet showing OS differences between these groups, our results from a relatively uniformly managed group of patients clearly suggest that the biology of the groups is important, independent of initial surgical management.

IDHwt is relatively uncommon in strictly defined grade II glioma, 9%(14 patients) in this series. Here, classic astrocytoma morphology predominates, but it remains for a much larger, well-annotated study of these tumors to demonstrate whether or not prognostically different molecular subtypes can be defined within this group and whether or not clinical and treatment factors influence their outcomes.

Despite the retrospective nature of the study, our cohort has the unique advantage of treatment uniformity and long-term follow-up. The majority of patients with >90% EOR were observed closely and received radiation therapy at first recurrence, while most patients with subtotal resection had adjuvant radiation. In this strictly defined grade II glioma cohort, we demonstrate that IDHmut, 1p19q codel, and ATRX loss, considered together, are prognostically useful. Molecular groups defined by combinations of these markers should be incorporated in the neuropathologic evaluation.

## MATERIALS AND METHODS

The Mayo Clinic Brain SPORE (Specialized Program of Research Excellence) Registry was queried to identify patients aged 16 years or older with a tissue diagnosis of WHO grade II O, OA, or A between January 1, 2003, and December 31, 2012. A total of 159 patients who received care at Mayo Clinic were identified with tissue available for review and appropriate consent. Seven patients had resection at Mayo Clinic Rochester for a WHO grade II glioma prior to 2003, dating 1992 at the earliest. Thirty-two patients had previous resection elsewhere dating to 1990 at the earliest. Slides from these previous resections were reviewed by a Mayo neuropathologist and diagnosed as WHO grade II glioma. Additional studies (IHC, 1p19q FISH) were obtained at Mayo as needed. Their pertinent clinical data was abstracted from the electronic medical record including: 1) extent of resection (EOR) as determined by surgeon and corroborated by treating oncologist and interpreting neuroradiologist, 2) dates of tumor progression as determined by treating oncologist, 3) and dates of most recent MRI documenting tumor stability. Institutional Review Board approval was obtained. Data from 37 patients have been previously published [4].

All cases were primarily diagnosed by a neuropathologist, applying strict uniform diagnostic criteria, including: low to moderate cellularity defined as cellularity of tumors in which cell density is such that background infiltrated parenchyma is easily recognizable even in low-intermediate power (x10–x20), in contrast to high cellularity in which cell density is such that tumor cells are tightly packed touching each other (intervening parenchyma is present but its recognition requires intermediate-high power resolution x20–x40; e.g. to recognize entrapped neurons); low mitotic index, which differs between astrocytic and oligodendroglial tumors. In WHO grade II astrocytoma, no mitotic activity should be seen, although a single mitosis was not considered sufficient for a grade III a designation [30]. In WHO grade II oligodendroglioma and oligoastrocytoma, mitotic index should be <6 per 10 high-power fields (HPF), a threshold we established and [31] has been widely used in the neuropathology community and referenced in the WHO 2007. Grade II astrocytoma (WHO) were typically low to moderately cellular tumors with no or, at most, a rare mitotic figure. Grade II oligodendroglioma and oligoastrocytoma (WHO) were low to moderately cellular tumors with <6 mitoses per 10 HPF. Neither microvascular proliferation nor necrosis were present [31]. With only three neuropathologists on service over the time period involved, one of which trained under the other two, diagnostic criteria and their application were very uniform, as shown by the high intraclass correlation coefficient (“substantial to almost perfect”) observed among two (of three) neuropathologists on all grading criteria in oligodendroglioma [31]. At the time of this study, histology review was conducted by a single neuropathologist (CG) to confirm the diagnosis and grade as well as to assess tumoral tissue markers. The following markers were evaluated in each case: p53 protein and ATRX expression by immunohistochemistry (IHC); IDH mutation status by IDH1 R132H immunohistochemistry followed by IDH1-IDH2 pyrosequencing in all IHC-negative cases, 1p19q status by FISH. Clinical testing of codel by FISH has been in use since 2003, while IDH1R132H IHC was implemented in 2011, and IDH1-IDH2 pyrosequencing in 2012. Immunohistochemistry for p53 protein has been routinely used for many years. Thus, within our grade II glioma cohort, 1p19q status was known in 128 cases, IDH mutation status in 37, and p53 expression had been tested in 71. Analysis was completed in all cases lacking one or more of the tissue markers (p53 expression, IDH status, and 1p19q status) if sufficient tissue was available from the primary tumor resection (p53 expression) and/or from subsequent tumor resections (IDH, ATRX and 1p19q status). Since 1p19q codeletion, IDH and ATRX mutation are early genomic alterations, typically shared between primary and recurrent tumors, testing of recurrent tumors should not have significantly affected the results of our series. 1p19q status was assessed in the primary tumor resection in 142 (of 151) cases (94%) and IDH status in 128 (of 155) cases (82%) while in remaining cases was assessed on tumor from subsequent resections. Mutations of IDH were evaluated by immunohistochemistry using the monoclonal antibody to IDH1R132H H09 and by pyrosequencing in immunonegative cases as recently described [32]. IDH1R132H IHC was scored as positive (consistent with IDH1 R132H mutation) or negative. IDH1-IDH2 pyrosequencing is designed to detect IDH1 R132C, R132G, R132H, R132L, R132P, and R132S and IDH2 R172G, R172K, and R172M mutations, while IDH1 R132V and R100Q and IDH2 R172S, R172T and R172W, which occur at very low frequency, are not detected. In 20 cases, testing was performed using a recently developed PCR assay for one-step detection of 12 IDH1/IDH2 mutations, which allows for identification of 12 IDH1-IDH2 mutations, including IDH1 R100 mut, three among which are specifically identified (IDH1 R132H, IDH1 R132C and IDH2 R172K), while the others are reported as “unknown” [33]. FISH for 1p19q was performed according to previously described methods [15]. IHC stain for p53 protein was performed according to standard techniques. Positive p53 staining was used as a surrogate for altered TP53 function as previously described [34]. Percentage of cells displaying strong nuclear staining was scored semi-quantitatively as 0 to 10% of tumor cell, 11 to 50%, 51 to 80%, or >80%.

ATRX expression was evaluated in 93 tumors, including all IDHmut-noncodel tumors with sufficient tumor tissue from either the original and/or a subsequent tumor surgery and a small number (*n* = 12) of 1p19q codel tumors. ATRX expression was assessed in the primary tumor resection in 75 (of 93) cases (81%) and in tumor from subsequent resections in remaining cases [35]. IHC was performed using the monoclonal antibody anti-ATRX (clone D5) 1:1000 dilution of primary antibody (Santa Cruz, catalog # sc-55584), performed on the Ventana BenchMark XT stainer with the following conditions: CC1 for 32 min as pretreatment, primary antibody incubation time of 16 minutes at 37°C, Optiview detection, followed by Ventana DAB, 8 minutes hematoxylin and 4 minutes Bluing Reagent. ATRX expression was either retained or lost in tumor cells (Figure 1). In a few cases (*n* = 6), the number of ATRX-negative cells seemed to be focally less than the number of atypical tumor cells identified morphologically and by IDH1 R132H IHC stain (5 cases were IDH1R132H IHC positive). These were areas in which vascular cells and neurons stained positively, excluding that the lack of stain could be artifactual. We originally tracked these cases as “partial ATRX loss, ” since when we reviewed the ATRX stain we were blind to the 1p19q results. We wanted to exclude that these cases may represent possible rare examples of “true mixed gliomas with molecular features of composite oligodendroglioma and astrocytoma [36, 37].” The cases were 1p19q intact and were grouped with the ATRX loss cases.

### Statistical analyses

Group comparisons of patient, surgical, and tumor characteristics were made using the chi-square test for categorical variables and the Kruskal-Wallis test for continuous variables. Cumulative survival probabilities were estimated using the Kaplan-Meier method. Cox proportional hazards regression was used to assess the association of patient, surgical, and tumor characteristics with overall and progression-free survival (PFS). All analyses included age, sex, histomorphologic diagnosis, extent of resection, radiation therapy, and chemotherapy as covariates. In all cases, *p*-values < 0.05 were considered statistically significant.
